# Pulmonary Congestion Assessed by Lung Ultrasound and Cardiovascular Outcomes in Patients With ST-Elevation Myocardial Infarction

**DOI:** 10.3389/fphys.2022.881626

**Published:** 2022-05-10

**Authors:** Diego Araiza-Garaygordobil, Luis A. Baeza-Herrera, Rodrigo Gopar-Nieto, Fabio Solis-Jimenez, Alejandro Cabello-López, Pablo Martinez-Amezcua, Vianney Sarabia-Chao, Héctor González-Pacheco, Daniel Sierra-Lara Martinez, José Luis Briseño-De la Cruz, Alexandra Arias-Mendoza

**Affiliations:** ^1^ Coronary Care Unit, Instituto Nacional de Cardiología “Ignacio Chávez”, Mexico City, Mexico; ^2^ Occupational Health Research Unit, Centro Médico Nacional Siglo XXI, Instituto Mexicano del Seguro Social, Mexico City, Mexico; ^3^ Department of Epidemiology, Johns Hopkins Bloomberg School of Public Health, Baltimore, MD, United States

**Keywords:** STEMI, lung ultrasound, pulmonary congestion, acute heart failure, heart failure

## Abstract

**Background:** Lung ultrasound (LUS) shows a higher sensitivity when compared with physical examination for the detection of pulmonary congestion. The objective of our study was to evaluate the association of pulmonary congestion assessed by LUS after reperfusion therapy with cardiovascular outcomes in patients with ST-segment Elevation acute Myocardial Infarction (STEMI) who received reperfusion therapy.

**Methods:** A prospective observational study including patients with STEMI from the PHASE-Mx study. LUS was performed in four thoracic sites (two sites in each hemithorax). We categorized participants according to the presence of pulmonary congestion. The primary endpoint of the study was the composite of death for any cause, new episode or worsening of heart failure, recurrent myocardial infarction and cardiogenic shock at 30 days of follow-up.

**Results:** A total of 226 patients were included, of whom 49 (21.6%) patients were classified within the “LUS-congestion” group and 177 (78.3%) within the “non-LUS-congestion” group. Compared with patients in the “non-LUS-congestion” group, patients in the “LUS-congestion” group were older and had higher levels of blood urea nitrogen and NT-proBNP. Pulmonary congestion assessed by LUS was significantly associated with a higher risk of the primary composite endpoint (HR: 3.8, 95% CI 1.91–7.53, *p* = 0.001). Differences in the primary endpoint were mainly driven by an increased risk of heart failure (HR 3.91; 95%CI 1.62–9.41, *p* = 0.002) and cardiogenic shock (HR 3.37; 95%CI 1.30–8.74, *p* = 0.012).

**Conclusion:** The presence of pulmonary congestion assessed by LUS is associated with increased adverse cardiovascular events, particularly heart failure and cardiogenic shock. The application of LUS should be integrated as part of the initial risk stratification in patients with STEMI as it conveys important prognostic information.

## Introduction

Pulmonary congestion is a powerful prognostic factor for the detection of adverse cardiovascular events, including death, in patients with STEMI (Killip and Kimball, 1967). In addition, the presence of pulmonary congestion increases the discriminatory capacity of scoring classifications such as Thrombolysis in Myocardial Infarction (TIMI) and Global Registry on Adverse Cardiovascular Events (GRACE) (Bedetti et al., 2010).

Lung ultrasound is a non-invasive, risk-free tool that has demonstrated to be superior when compared with physical examination for the detection of pulmonary congestion due to a higher sensitivity (Gopar -Nieto et al., 2019). However, the association between the degree of pulmonary congestion detected by LUS and cardiovascular outcomes in patients with STEMI has not been completely elucidated.

The objective of our study was to evaluate the association of pulmonary congestion assessed by LUS with cardiovascular outcomes in patients with STEMI.

## Materials and Methods

### Study Population and Design

The study population derives from PHASE-Mx Study “PHArmacoinvasive Strategy vs. primary PCI in STEMI: a prospective registry in a largE geographical area” (www.clinicaltrials.gov NCT03974581); the description, design, scope and detailed results of PHASE-MX study have been published elsewhere (Baeza -Herrera et al., 2020; Araiza-Garaygordobil et al., 2021a). Briefly, this prospective observational study was conducted from March 2018 to March 2020 and included adults older than 18 years-old diagnosed with STEMI, who received reperfusion treatment in the first 12 h since symptoms onset. Patients with previous diagnosis heart failure (HF), pulmonary diseases, >12 h from symptom onset to treatment, unknown ischemic time, those who did not receive acute reperfusion, with in- hospital STEMI from other causes, or with a discharge diagnosis other than STEMI were excluded. Lung ultrasound was performed during the first 24 h from symptom onset and after reperfusion therapy. The protocol received local research and ethics committee approval (PT-19-109) and complies with the principles of the Declaration of Helsinki. Written informed consent was obtained from all patients prior to study inclusion.

### Lung Ultrasound Technique

**FIGURE 1:**
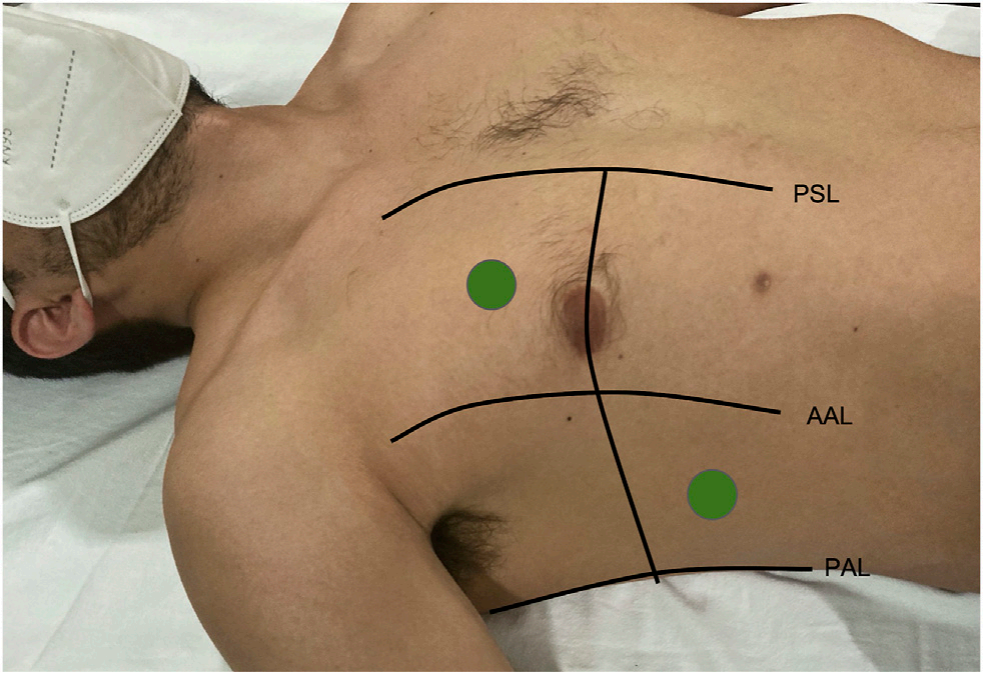
Schematic lung ultrasound technique.

LUS was performed using a portable device equipped with a 3.8 MHz phased array transducer (VScan® Dual Probe; GE Healthcare, Chicago, IL, United States) during the first 24 h of hospitalization and after reperfusion therapy. LUS was recorded in four thoracic sites, two sites in each hemithorax (4-point method) (Figure 1) with the transducer in axial orientation and at 18 cm imaging depth with the patient in semi- recumbent position, following expert panel recommendations (Platz et al., 2019). The number of B-lines reported was the higher sum of B-lines visualized in each site during a 3-s clip.

### Definition of Pulmonary Congestion

For analytical purposes, participants who had at least one bilateral quadrant with ≥3 B-lines were considered within the “LUS-congestion” group; the rest of the participants were considered within the “non-LUS-congestion” group.

### Outcomes

The patients were followed-up by a pre-specified visit. As the follow-up was short, no losses were recorded. The primary endpoint was the composite of death for any causes, new episode or worsening of HF, recurrent myocardial infarction (MI) and cardiogenic shock at 30 days of follow-up. HF was defined as the onset or worsening of symptoms such as dyspnea, edema, orthopnea or initiation/increase of intravenous diuretics dose. Recurrent MI was defined according to the 2017 Cardiovascular and Stroke Endpoint Definitions for Clinical Trials (Hicks et al., 2018). Cardiogenic shock was defined as systolic blood pressure lower than 90 mm Hg or use of vasopressors with signs of poor peripheral perfusion, secondary to low cardiac output (assessed by echocardiography) at any time during hospitalization.

### Sample Size Estimation

Based on an interim analysis after the enrollment of the first 60 patients, considering an estimated incidence of the primary endpoint to be around 10% at 30 days follow-up, an expected absolute difference of the occurrence of the primary endpoint between groups of 15%, and accounting for a power (1-β) of 80% and an alpha level of 0.05%, a sample size of 194 patients was calculated. Accounting for 10% potential losses during follow-up, a final sample of 214 patients was estimated.

### Statistical Analysis

Categorical variables were expressed as relative and absolute frequencies. Continuous variables were expressed as means (standard deviation) or medians (interquartile range). Covariates were compared between congestion groups using Student’s t test, Mann-Whitney’s U test and Chi square test, as appropriate. Time to occurrence of the primary outcome was evaluated with Kaplan-Meier curves, log-rank test and Cox proportional hazards models. We used a multivariate model adjusted for age, sex and NT-proBNP, and it was tested with the variables that showed significance after univariate analysis. Inter and intra-observer agreement in LUS interpretation was evaluated with intraclass correlation coefficients and is included in the Supplementary Appendix S1. A two-sided level of 0.05 was considered significant. Stata 14 (STATA corp) was used for all analyses, and results were reported following STROBE diagram and checklist (Von Elm et al., 2007).

## Results

**FIGURE 2:**
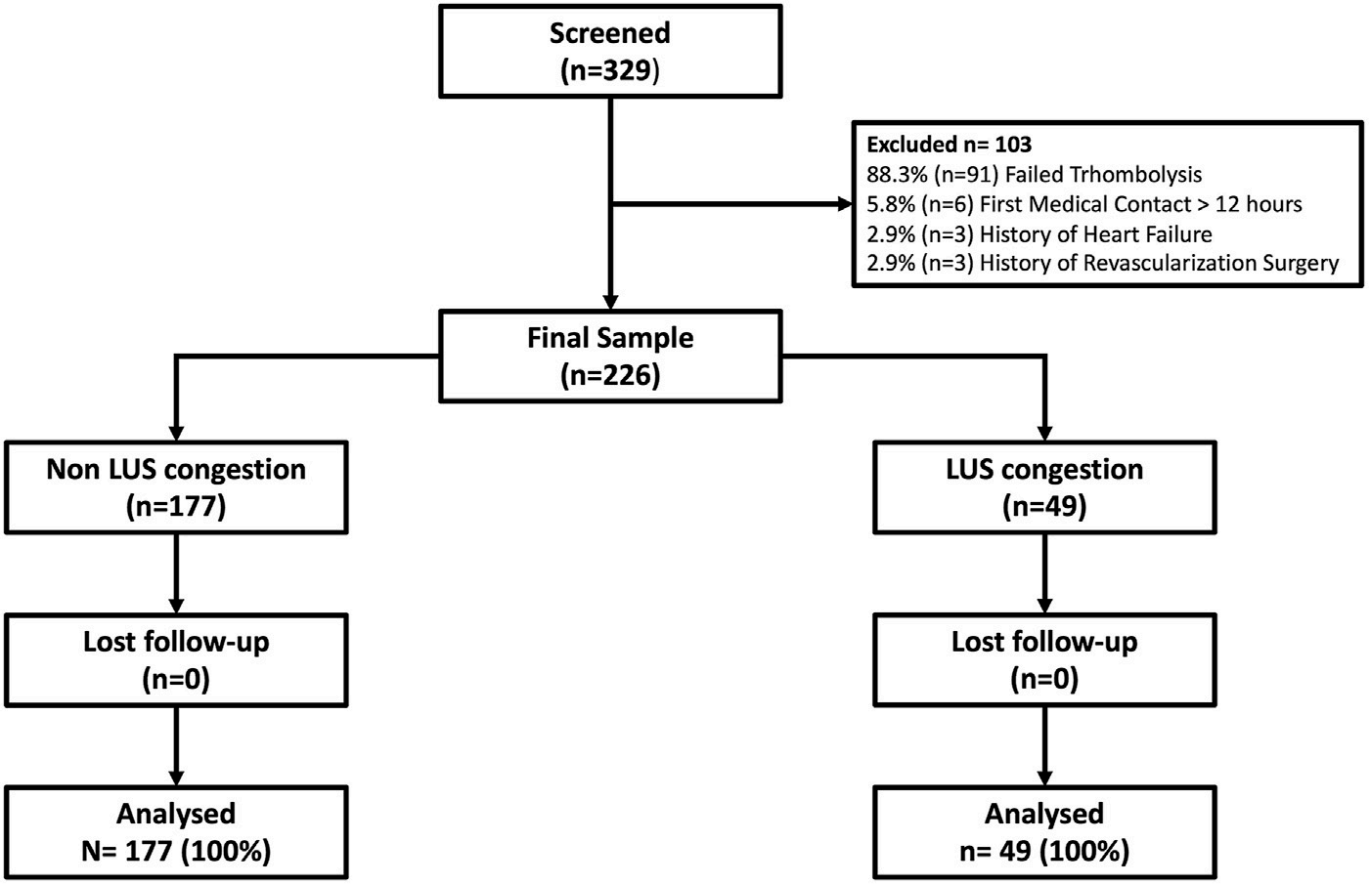
Flowchart of the sampling process.

From the total population included in the PHASE-Mx study, LUS was performed only in 329 of which 103 were excluded due to the following specific causes: 91 patients due to failed thrombolysis, six patients due to first contact time greater that 12 h, three patients due to previous heart failure, and three patients due to previous revascularization surgery. Therefore, the final analytic sample consisted of 226 patients. Baseline laboratory characteristics were taken at hospital admission, while lung ultrasound was performed at any time after revascularization and within 24 h from the symptom onset. There were 49 (21.6%) patients classified within the “LUS-congestion” group and 177 (78.3%) within the “non-LUS-congestion” group (Figure 2). Baseline characteristics of the study population stratified by presence or absence of LUS congestion are summarized in Table 1. Compared with patients in the “non-LUS-congestion” group, patients in the “LUS-congestion” group were older (61.51 vs. 57.23 years, *p* = 0.015) and had higher levels of blood urea nitrogen (21.22 vs. 17.75 mg/dl, *p* = 0.023) and NT-proBNP (3,488.01 vs.1377.04 pg/ml, *p* < 0.001).

**TABLE 1:** General characteristics of the population according to pulmonary congestion evaluated by LUS.

	Overall	No LUS congestion *n* = 177	LUS congestion *n* = 49	*p* Value
Demographic characteristics				
Male, (%)	202 (89.3)	162 (93.64%)	40 (85.11%)	0.058
Age, (IQR)	59.9 (50–65)	57.23 (49–64)	61.51 (56–66)	0.015
Diabetes, (%)	66 (29.33)	53 (30.11%)	13 (26.53%)	0.0626
Hypertension, (%)	100 (55.25%)	74 (41.81%)	26 (53.06%)	0.160
Dyslipidemia, (%)	39 (17.26%)	33 (18.64%)	6 (12.24%)	0.294
Current smokers, (%)	109 (48.23)	87 (49.15%)	22 (44.90%)	0.598
Ever smokers, (%)	31 (13.72%)	24 (13.56%)	7 (14.29%)	0.896
Obesity, (%)	48 (21.24%)	40 (22.60%)	8 (16.33%)	0.342
Previous PCI, (%)	12 (5.31%)	11 (6.21%)	1 (2.04%)	0.222
Previous CABG, (%)	2 (0.88%)	1 (0.56%)	1 (2.04%)	-
Admission characteristics				
Heart Rate (IQR)	77.63 (67.89)	76.70 (65–89)	81.02 (70–89)	0.111
Respiratory Rate (IQR)	18.33 (16–19)	18.10 (16–18)	19.14 (16–20)	0.422
Systolic Blood Pressure (IQR)	131.30 (114–146)	131.02 (115–145)	132.30 (111–149)	0.751
Diastolic Blood Pressure (IQR)	81.65 (70–90)	81.61 (70.90)	81.77 (70–92)	0.952
Glucose (normal range 70–105 mg/dl) (IQR)	191.54 (124–225)	186.72 (124–216)	208.93 (124–273)	0.172
Creatinine (normal range 0.5–0.9 mg/dl) (IQR)	1.23 (0–1)	1.23 (0–1)	1.22 (0–1)	0.972
BUN (normal range 6–20 mg/dl) (IQR)	17 (14–21)	16 (14–20)	19 (15–25)	0.006
LVEF (SD)	44.68 (11.95)	45.39 (11.7)	42.57 (12.3)	0.154
Troponin (normal range 3–14 pg/ml) (IQR)	24.83 (0–50)	23.92 (0–39)	28.10 (0–59)	0.408
NT-ProBNP (normal range 15–450 ng/ml) (IQR)	541.5 (125–2209)	384 (113–1371)	1701 (407–4025)	0.001
TIT (min) (IQR)	270 (171–382)	261 (155–367)	280 (190–420)	0.169
Reperfusion method				
Thrombolysis, (%)	92 (40.71)	77 (43.50)	15 (30.61)	0.104
DNT (IQR)	79.93 (25–90)	81.96 (58–99.2)	73.92 (23–112.5)	0.262
PCI, (%)	134 (59.29)	100 (56.50)	34 (69.39)	0.104
DBT (IQR)	90.03 (59–99)	85.96 (58–99.2)	100.2 (60–102.2)	0.262
Prognostic scales				
Killip-Kimball Class				<0.001
Class I	139 (61.5%)	118 (66.6%)	21 (42.8%)	
Class II	76 (33.62%)	56 (31.6)	20 (40.8%)	
Class III	4 (1.76%)	2 (1.12%)	2 (9.8%)	
Class IV	7 (3.09%)	1 (0.56%)	6 (12.2%)	
TIMI (IQR)	3.42 (3.12–3.73)	3.25 (2.93–3.5)	4.08 (3.25–4.91)	0.028
GRACE (IQR)	121.05 (166.21–125.91)	117.28 (112–122.41)	135.02 (123.7º–146.96)	0.003
CRUSADE (IQR)	27.65 (25.4–29.8)	25.92 (23.85–27.99)	33.73 (27.14–40.32)	0.003
Angiographic characteristics				
Culprit artery				0.465
LMCA, (%)	9 (4.57)	6 (3.90)	3 (6.98)	
LADA, (%)	80 (38.96)	60 (38.96)	20 (46.51)	
Circumflex artery, (%)	21 (10.66)	16 (10.39)	5 (11.63)	
RCA, (%)	87 (44.16)	72 (46.75)	15 (34.88)	
No reflow phenomenon, (%)	29 (20.71%)	21 (20%)	8 (22.86%)	0.718

IQR, interquartile range; PCI, percutaneous coronary intervention; CABG, coronary artery bypass graft; BUN, blood urea nitrogen; LVEF, left ventricular ejection fraction; SD, standard deviation; NT-ProBNP, N-terminal pro-B type natriuretic peptide; TIT, total ischemic time; DNT, door needle time; DBT, door balloon time; TIMI, thrombolysis in myocardial infarction; GRACE, global registry on acute coronary events; CRUSADE, Can Rapid risk of major bleeding of Unstable angina patients suppress Adverse outcomes with Early implementation of the ACC/AHA, guidelines; LMCA, left main coronary artery; LADA, left anterior descending artery; RCA, right coronary artery.

Patients in the LUS-congestion group had higher TIMI, GRACE and CRUSADE scores compared to patients in the non-LUS-congestion group. The total ischemic time was not different between patients with and without pulmonary congestion (median time: 316 vs. 282 min, respectively, *p* = 0.169). A higher proportion of patients with LUS congestion (55.3%) were classified as Killip-Kimball class (KKC) >I compared with patients in the non-LUS- congestion group (30.4%) (*p* < 0.001).

### Outcomes

**FIGURE 3:**
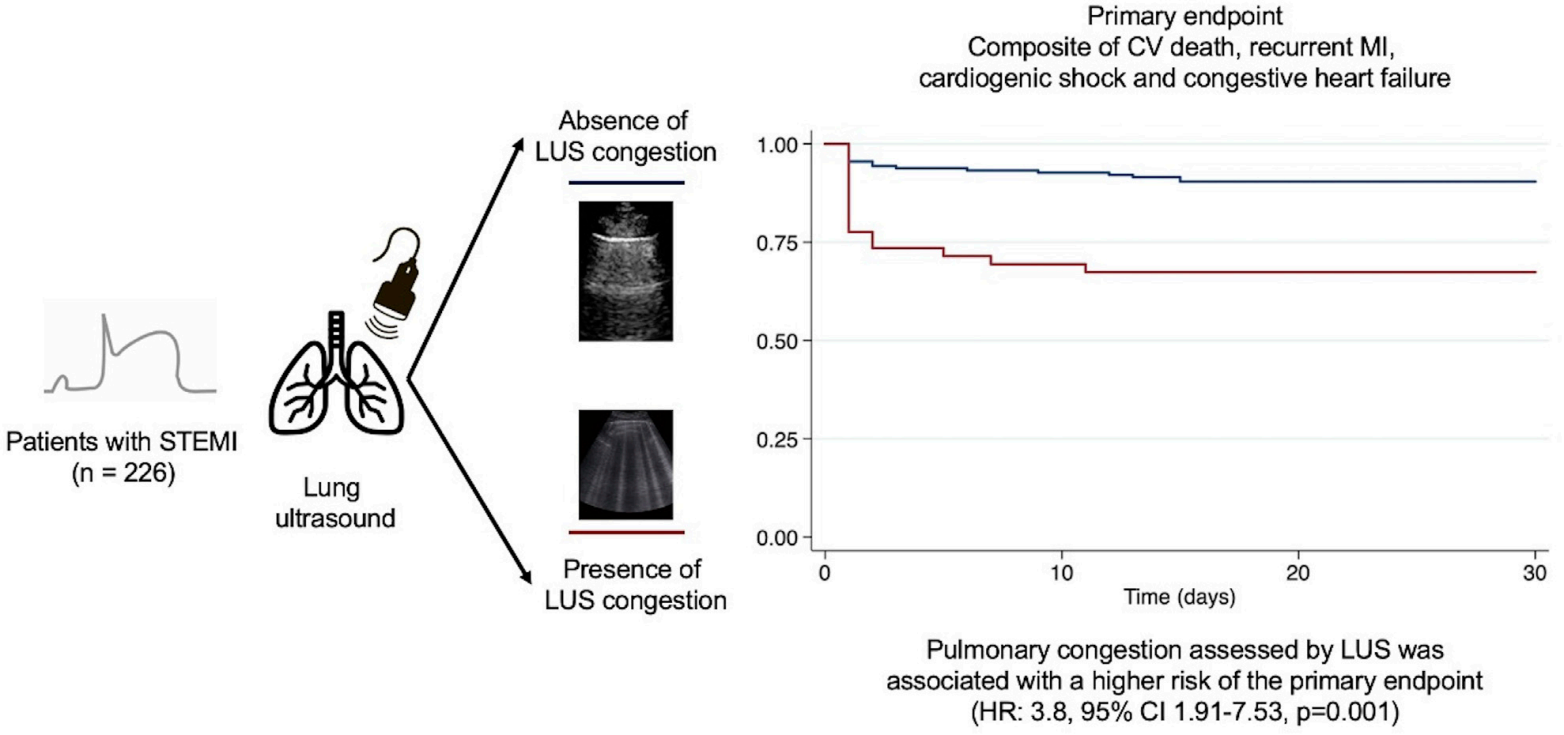
Central figure. Kaplan Meier estimates for the primary endpoint in patients with STEMI and pulmonary congestion assessed by lung ultrasound.

**FIGURE 4:**
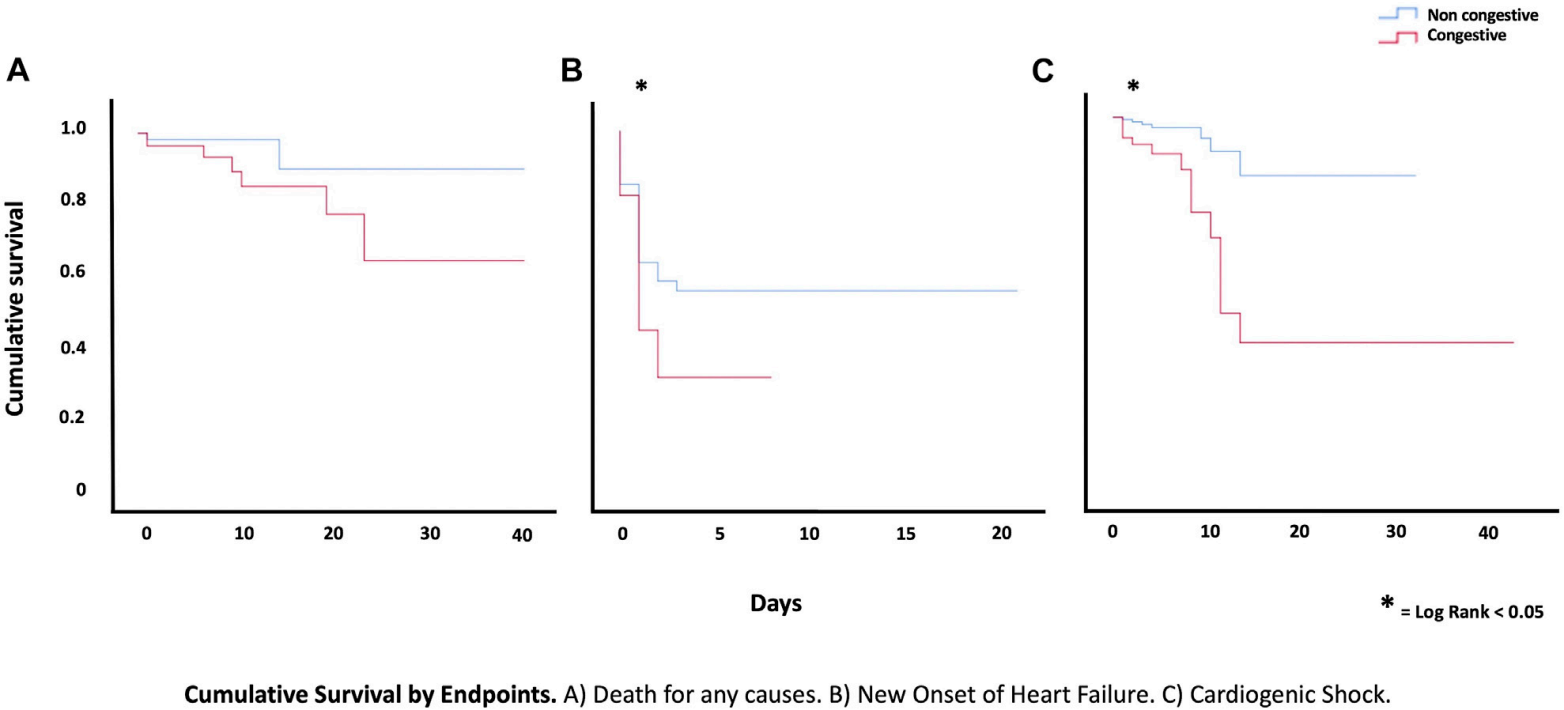
Cumulative Survival by Endpoints. **(A)** Death for any causes. **(B)** New Onset of Heart Failure **(C)** Cardiogenic Shock.

Overall, 14.60% (*n* = 33) of patients presented the primary outcome after 30 days of follow-up. Pulmonary congestion assessed by LUS was significantly associated with a higher risk of the primary composite endpoint (HR: 3.8, 95%CI 1.91–7.53, *p* = 0.001) (Figure 3). Differences in the primary endpoint were mainly driven by an increased risk of heart failure (HR 3.91; 95%CI 1.62–9.41, *p* = 0.002) and cardiogenic shock (HR 3.37; 95%CI 1.30–8.74, *p* = 0.012). (Figure 4). No significant differences were noted in the rates of reinfarction and cardiovascular mortality Table 2 shows the proportion of events according to the presence or absence of LUS- congestion.

**TABLE 2:** Outcomes in patients with ST-segment elevation myocardial infarction and pulmonary congestion assessed by LUS.

	Overall	Without congestion N = 177	Congestion *n* = 49	*p*
Primary Outcome, n (%)	34 (15%)	17 (9.6%)	17 (34.69%)	0.001
Heart Failure, n (%)	20 (8.85%)	10 (5.65%)	10 (20.41%)	0.001
Reinfarction*, n (%)	2 (0.56%)	1 (0.56)	1 (2.04)	0.387*
Death for any causes, n (%)	11 (4.8)	6 (3.39%)	5 (10.2%)	0.063*
Cardiogenic shock, n (%)	17 (7.52%)	9 (5.08%)	8 (16.33%)	0.008

*Fisher’s exact test. IQR, interquartile range (Q1–Q3); LUS, lung ultrasound.

After multivariable analysis, the association of LUS-congestion and the occurrence of the primary endpoint remained statistically significant and exceeded the effect of other clinically relevant variables such as age, diabetes, TIMI and GRACE scores and NT-proBNP (Table 3). Finally, the sensitivity, specificity and area under the ROC curve to predict the composite primary outcome were 60, 77.2 and 73%, respectively. The incremental prognostic value of LUS-congestion (when compared with KKC) as assessed by integrated discrimination improvement (IDI) was 6.0% (95%CI 4.3–8.7, *p* < 0.001).

**TABLE 3:** Predictors of the primary outcome in Cox regression analysis.

	Univariate		Multivariate	
	HR CI 95%	*p*	HR CI 95%	*p*
Age >60 years	3.80 (1.91–7.53)	0.001	1.82 (0.79–4.19)	0.159
Diabetes Mellitus	3.05 (1.53–6.05)	0.001	2.62 (1.28–5.33)	0.008
TIMI >4 points	5.32 (2.30–12.32)	0.001	2.63 (1.03–6.69)	0.042
GRACE score >140	2.86 (1.44–5.66)	0.003	1.53 (0.73–3.23)	0.255
Pulmonary Congestion (LUS)	3.80 (1.91–7.53)	0.001	3.17 (1.52–6.62)	0.002
NT-ProBNP	3.86 (1.79–8.32)	0.001	1.61 (0.68–3.80)	0.277

GRACE, global registry on acute coronary events; HR, hazard ratio; LUS, lung ultrasound; TIMI, thrombolysis in myocardial infarction.

## Discussion

In the present study, pulmonary congestion assessed by LUS in patients with STEMI was associated with a higher frequency of adverse cardiovascular events, particularly acute HF and cardiogenic shock.

The interest in the use of LUS as a non-invasive tool for semi-quantification of pulmonary congestion has grown in recent years (Platz et al., 2017; Picano et al., 2018). LUS has demonstrated to be superior in the detection of pulmonary congestion in patients with HF, showing a higher sensitivity when compared with physical examination or chest X-ray (Pivetta et al., 2019; Araiza-Garaygordobil et al., 2021b). Furthermore, studies including patients with chronic or acute decompensated HF have demonstrated that LUS derived B- lines have an important prognostic role for the detection of HF-derived events, such as rehospitalizations or cardiovascular mortality (Miglioranza et al., 2017; Rivas-Lasarte et al., 2019; Araiza-Garaygordobil et al., 2020). This prognostic role exceeds that of other commonly used congestion evaluation parameters such as clinical examination or concentrations of natriuretic peptides (Miglioranza et al., 2013).

Acute HF after MI is a potentially serious complication that increases mortality. Detection of signs of HF after MI allows the identification of a subgroup of patients with worse prognosis. Reduced left ventricular ejection fraction, increased concentrations of natriuretic peptides, increased pulmonary capillary wedge pressure (using a pulmonary flotation catheter), and physical examination showing signs of HF (lung crackles, presence of a third heart sound, jugular vein distention or peripheral oedema) have all been associated with increased hospital mortality after MI (Platz et al., 2017; Öhman et al., 2018; Ye et al., 2019). LUS may complement the findings of the aforementioned techniques with additional advantages such as low cost, bedside availability and no associated risks. Recently, a prospective observational study (Araujo et al., 2020) documented the prognostic ability of admission LUS in 215 patients with STEMI. The investigators reported an area under the ROC curve of 0.89 for in-hospital mortality and a 0.18 net reclassification improvement over the KKC. It is worth mentioning that absence of pulmonary congestion detected by LUS implied a negative predictive value for in-hospital mortality of 98.1%. Likewise, our study shows consistent results, with an increased risk of adverse outcomes seen in those patients showing LUS congestion.

There are some limitations in our study. One of the most important limitations is that our study may be influenced by selection bias. As our Institute is a reference center, it is possible that some patients, who were unable to be transferred because of instability or who could have died before reaching our center, were not registered in our study. This would explain a relatively low frequency of some risk factors of adverse events, manifested by the low frequency of stage III or IV of the KKC, and lower TIMI score values. There is a small difference, with no statistical significance, in the pulmonary congestion group, as more received primary PCI. This could be related to the administration of contrast; unfortunately, we do not have the amount of contrast administered in each study. Although there is a difference mortality, it is not statistically significant, which may be determined by the relatively small sample size.

Lung ultrasound was performed after reperfusion therapy, and although the type of reperfusion strategy is balanced between both groups, we believe it may be one of the factors that influenced the limitation to predict mortality. To date, we do not know the influence of timely reperfusion on the presence and variation of the number of B-lines in STEMI patients. Nonetheless, a statistically significant association between the presence of B-lines in STEMI and major adverse endpoints during hospital stay was found, which strengthens the importance of pulmonary congestion among patients with STEMI, even after adequate reperfusion therapy was received.

LUS is a readily available, risk-free diagnostic tool that predicts adverse cardiovascular events in patients with STEMI. The application of this technique should be integrated as part of the initial risk stratification protocol for all patients with suspected STEMI as it conveys important prognostic information.

## Data Availability

The raw data supporting the conclusion of this article is available under reasonable request to the corresponding author.

## Abbreviations

LUS, Lung ultrasound; KKC, Killip-Kimball class; STEMI, ST-segment elevation myocardial infarction; HF, heart failure; MI, myocardial infarction; TIMI, Thrombolysis in Myocardial Infarction; GRACE, Global Registry on Adverse Cardiovascular Events.
